# Supplementary material for: Cuban Sugarcane Wax Alcohol Supplementation Prevents Brain and Eye Damages of Zebrafish Exposed to High-Cholesterol and High-Galactose Diet for 30 Weeks: Protection of Myelin, Cornea, and Retina
Source: Antioxidants (Basel). 2025 Dec 3;14(12):1453. doi: 10.3390/antiox14121453 (PMC12729879; doi:10.3390/antiox14121453)
Supplement: Supplementary file 1 [file antioxidants-14-01453-s001.zip › Table S1, Figures S1-S4, and Secitons S1-S2.pdf]

# Supplementary Material

## Supplementary Table S1

RAYDEL
Health Confidence

**Raydel Australia Pty Ltd**
Level 1, building 1, 9-15 Chilvers Rd.
Thornleigh NSW 2120 Australia
Tel +61 2 9480 1300
Fax +61 2 9480 1399
A.B.N. 45 054 555 903
www.raydel.com.au
info@raydel.com.au

**Product name: Policosanol**

Batch #: 310030324

Date of Manufacture: 12/03/2024

| Parameter | Results | Approved Limits |
|---|---|---|
| Color | Complies | Off white to cream |
| **Identity and Purity*** | | |
| 1-tetracosanol ($C_{24}$) | 0.05 % | 0.00 – 2.0 % |
| 1-hexacosanol ($C_{26}$) | 3.34 % | 3.0 –10.0 % |
| 1-heptacosanol ($C_{27}$) | 0.82 % | 0.1 - 3.0 % |
| 1-octacosanol ($C_{28}$) | 60.02 % | 60.0 –70.0 % |
| 1-nonacosanol ($C_{29}$) | 0.55 % | 0.1- 2.0 % |
| 1-triacontanol ($C_{30}$) | 14.96 % | 10.0 – 15.0% |
| 1-dotriacontanol ($C_{32}$) | 8.24 % | 5.0 - 10.0 % |
| 1-tetratriacontanol ($C_{34}$) | 2.39 % | 0.1 – 5.0 % |
| **Total (Purity*)** | **90.37 %** | **≥ 90 %** |
| **Other quality specifications** | | |
| **Melting temperature** | 81.3-83.0 °C | 78.0 – 83.0 °C |
| **Loss on drying** | 0.54 % | ≤ 1.0 % |
| **Residue of ignition** | 0.72 % | ≤ 0.85 % |
| **Heavy metals (Pb, Cd, Hg)** | <0.000015 % | ≤ 0.001 % |
| **Sodium content** | 86.34 ppm | ≤ 100 ppm |
| **Potassium content** | 2905.41 ppm | ≤ 4500 ppm |
| **Residual solvents** | | |
| **Acetone** | ≤ 0.03 | ≤ 0.03 g/kg |
| **Hexane** | ≤ 0.005 | ≤ 0.005 g/kg |
| **Microbiological content** | | |
| **Total Aerobic Microbial Count** | ≤10 | ≤$10^3$ per g |
| **Yeast and mould** | ≤10 | ≤$10^2$ per g |
| **Enterobacteria or Coliform count** | ≤10 | ≤$10^2$ per g |
| ***Staphylococcus aureus, Pseudomonas aeruginosa, Escherichia* coli, Candida albicans** | Absent | Absent in 1 g |
| ***Salmonella sp*** | Absent | Absent in 10 g |
| Observations: | | |
| **References:** | * Manufacturer GC validated method, purity expressed as the total of high molecular weight alcohols | |

**Note about storage conditions**: No special storage conditions are required. The substance has a shelf life of 5 years stored under ambient conditions of climatic Zones IV or II, as demonstrated in stability studies performed according to ICH guidelines.

Approved (_X_) Released (_X_) Rejected (___)

*This COA is it reproduced from supplier's COA*

**Table S1:** Certificate of analysis and the composition of the used policosanol

## Supplementary Material

## Section S1.

### 1. Quantification of blood lipoprotein profile hepatic function biomarkers AST and ALT

The plasma total cholesterol (TC) and triglycerides (TGs) were determined using commercial assay kits (cholesterol, T-CHO, and TGs, Cleantech TS-S; Wako Pure Chemical, Osaka, Japan) as per the method suggested by the suppliers. In brief, 5 μL serum was mixed with 200 μL reaction mixture (supplied with a commercial assay kit) for the TC analysis. The content was incubated at 37°C for 10 min, resulting in a red-colored product quantified by adsorption at 490 nm (Microplate reader, Bio-Rad, Hercules, CA, USA).

Similarly, 5 μL serum was mixed with a 200 μL of TGs-specific reaction mixture (supplied with a commercial assay kit) for TGs analysis. The content was incubated for 10 min at 37°C, and the formed colored product was quantified by taking adsorption at 490 nm.

For HDL-C analysis, serum was mixed in an equal ratio with the separation solution (supplied with a commercial assay kit), followed by centrifugation at 3,000 rpm for 10 min. The supernatant (20 μL) was collected and blended with a 200 μL reaction mixture (supplied with a commercial assay kit). After 10 min incubation at 37°C, red color intensity corresponding to HDL-C was quantified by taking absorption at 490 nm.

The commercial diagnostic kit (Asan Pharmaceutical, Hwasung, Republic of Korea) was used to quantify aspartate transaminase (AST) and alanine transaminase (ALT) levels in the plasma, following the instructions suggested by the manufacturers. Briefly, 5 μL of plasma was combined with 250 μL of either AST or ALT-specific solution, as supplied in the diagnostic kit. Following a 30 min incubation for AST or 60 min incubation of ALT at 37°C, the mixture was then blended with 250 μL of the respective coloring reagent (AST or ATL-specific, provided in the diagnostic kit). After a subsequent 20 min incubation at room temperature, 250 μL of 0.4 N NaOH was introduced to halt the reaction. Finally, the AST and ATL were quantified by measuring absorbance at 490 nm.

## Section S2.

### 2. Malondialdehyde (MDA) and ferric ion reduction (FRA) activity

The blood malondialdehyde (MDA) level was quantified by mixing plasma sample (20 μL, equivalent to 1 mg/mL protein) with trichloroacetic acid (50 μL, 0.2 mg/μL) and thiobarbituric acid (100 μL, 6.7 μg/μL). Following a 10-min incubation at 95 °C, the absorbance at 560 nm was recorded.

To assess ferric ion reduction (FRA) capacity, 20 μL of the plasma (1 1 mg/mL equivalent protein) was mixed with 180 μL of FRA reagent (prepared by blending 10 mL of acetate buffer (0.2M, pH 3.6) with 1.25 mL each of 2,4,6-tripridyl-S triazin (10 mM) and $FeCl_3$ (20 mM). After incubating the mixture at RT for 60 min, absorbance was measured at 593 nm. The results were quantified in μM ferric equivalents based on a ferrous sulfate standard curve.

**Supplementary Figure S1**

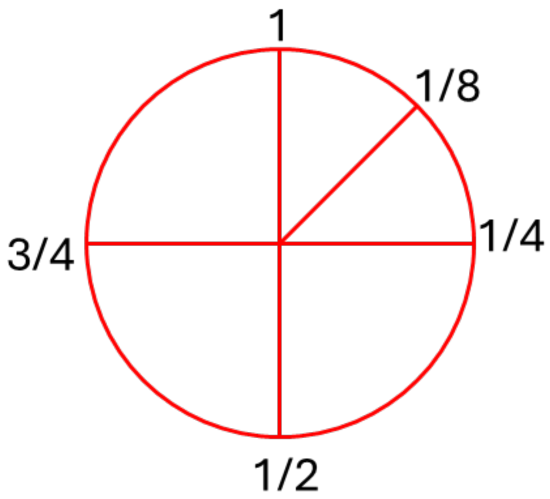


**Supplementary Figure S1:** Circular scale (developed by Cho & Ashu) to define the clouding/opacity area in the eyes of zebrafish. The numerical values in the circular scale representing the covered area.

**Supplementary Figure S2**

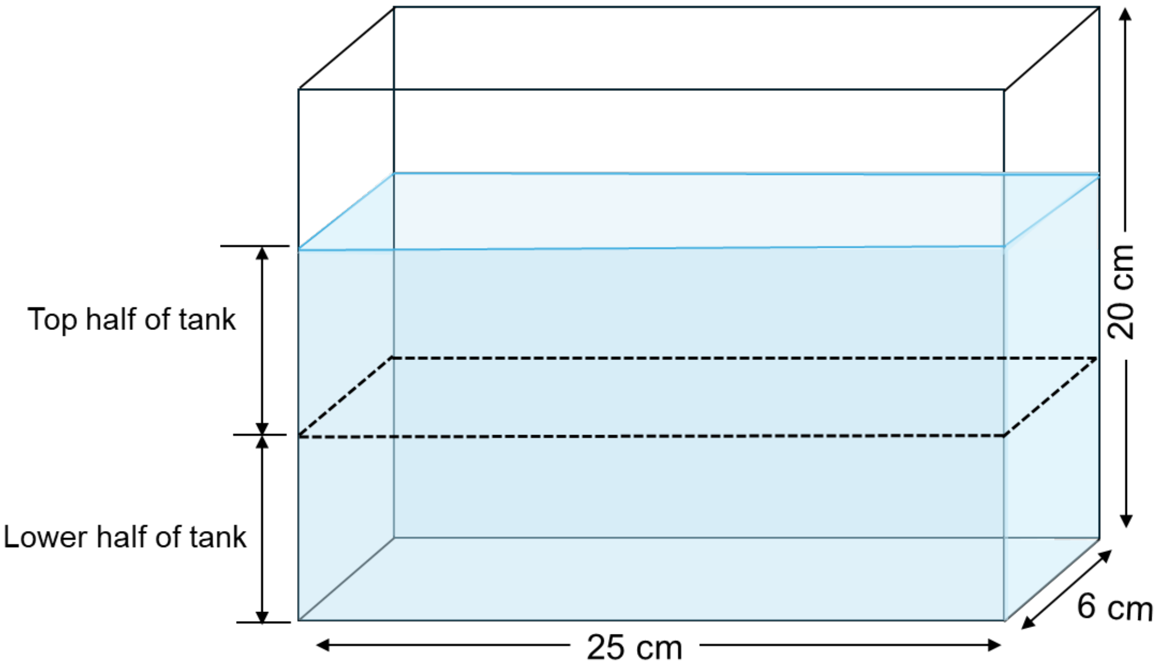


**Supplementary Figure S2:** Ray diagram of the open field water tank divided into two sectors (i.e., top half and lower half) using a dotted horizontal line.

**Supplementary Figure S3.**

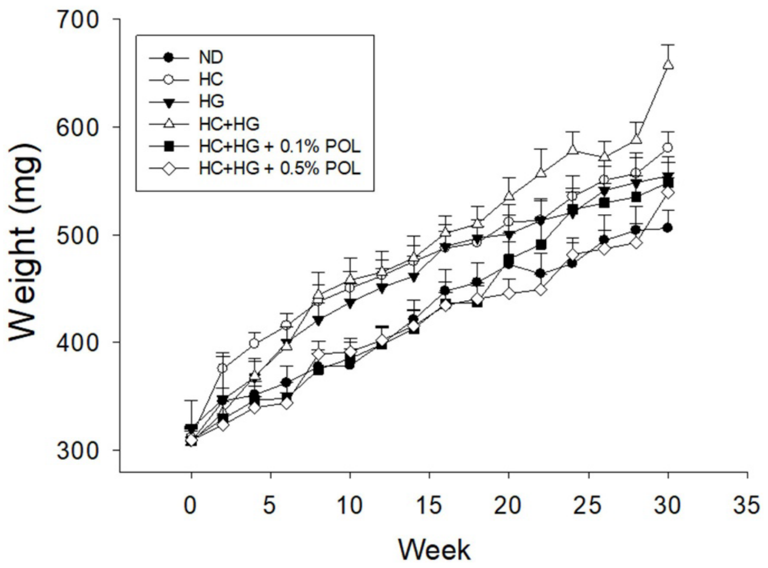


**Supplementary Figure S3.** Time dependent (0-30 weeks) changes in the body weight of zebrafish in different groups consuming the specialized diet. Abbreviations- ND: normal diet, HC: high cholesterol diet, HG: high galactose diet, HC+HG: high cholesterol+high galactose diet, 0.1% POL and 0.5% POL represent HC+HG diet supplemented with 0.1% and 0.5% policosanol.

**Supplementary Figure S4.**

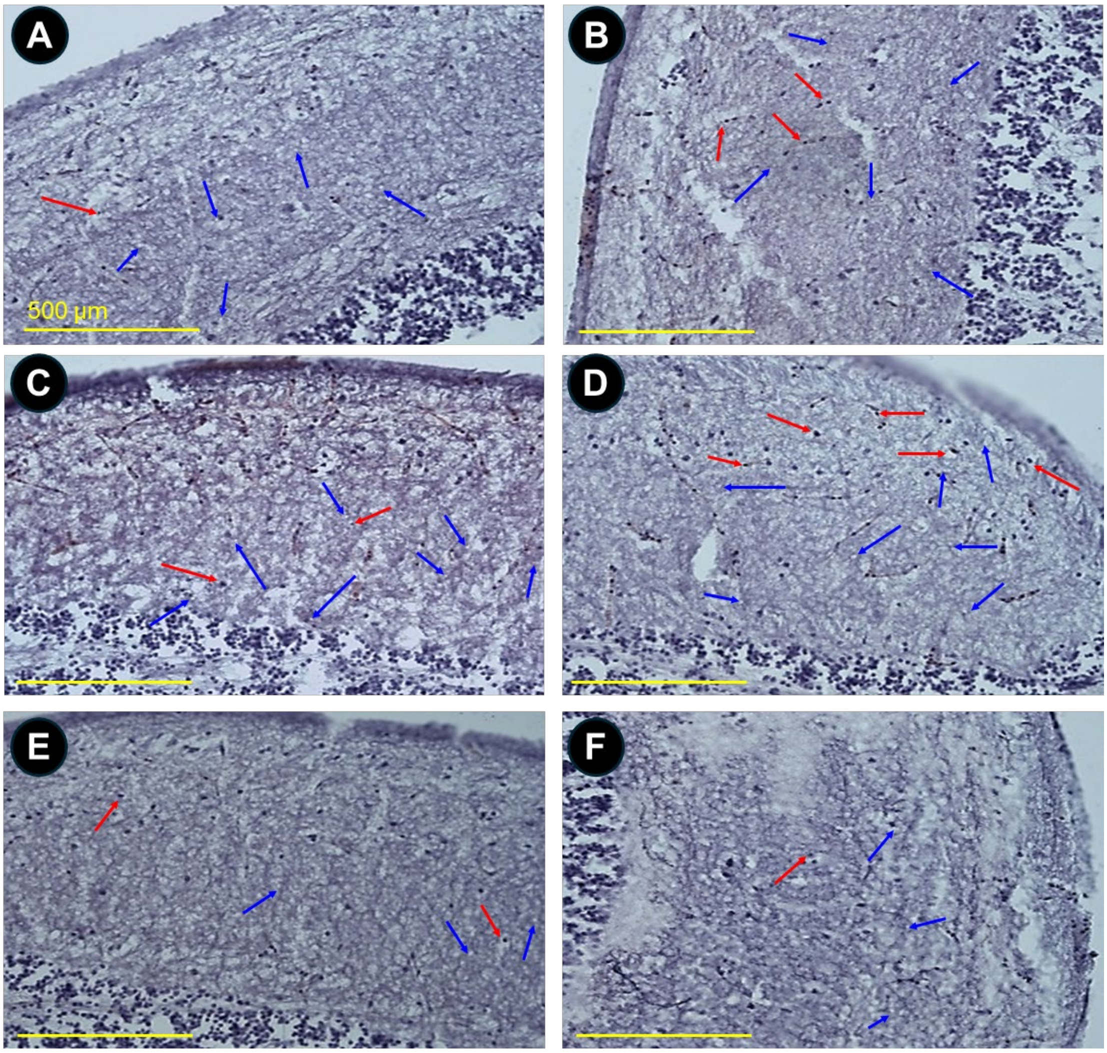


**Supplementary Figure S4.** A digitally magnified view of the H&E brain section. (A), (B), (C) and (D) represent the magnified view of the brain section obtained from ND, HC, HG, HC+HG, 0.1% POL and 0.5%POL supplemented group, respectively. Abbreviations- ND: normal diet, HC: high cholesterol diet, HG: high galactose diet, HC+HG: high cholesterol+high galactose diet, 0.1% POL and 0.5% POL represent HC+HG diet supplemented with 0.1% and 0.5% policosanol.
